# Case of Disease of the Heart

**Published:** 1814-10

**Authors:** John P. Batchelder


*For the Medical and Physical Journal.*

### *Case of Disease of the Heart; by* JOHN P. BATCHELDER, M.D.

THE patient was an infant four months old. From the birth it had been troubled, at times, with a difficulty of breathing, palpitations of the heart, and an increasing cough. Its breathing, when laborious, was accompanied with a peculiar sound. It could never bear an erect or sitting posture. The position in which it experienced the most ease was on the right side, partly inclined on the face, or directly on the face, with the head and shoulders incurvated. At the age of two months, our little patient was assailed with a most troublesome and obstinate cutaneous eruption, which might be considered almost epidemic in this part of the country, during the latter part of winter and spring. About a fortnight before he died, the eruption began to go off; and at the time of his death it had almost entirely disappeared, leaving only its sequelæ behind. On Tuesday all the symptoms were greatly aggravated; Wednesday they were very alarming, and continued to increase, with some intervals of ease, until Saturday about noon, when the child expired. During the paroxysms of distress, it was painful to behold him. At times the temperature of the body, and of the head in particular, was considerably increased; but, for the most part, the body and extremities were cold, and covered with a clammy sweat. He was frequently affected with syncope, which sometimes appeared more like extreme prostration of strength, than real fainting. At other times he was affected with agitations, which very much resembled convulsions. On Sunday the body was examined. We found some water in the ventricles of the brain, more than usual, I should conjecture; the exact quantity, however, could not be ascertained, for reasons soon to be mentioned. The blood-vessels were turgid; and there were some slight appearances of the cream-like matter, which is the result of inflammations about the brain. It is to be regretted that a nice dissection of the brain could not be made, on account of the derangement that organ suffered, in consequence of the violence we were obliged to use in removing the cranium, which adhered so firmly to the dura mater at the sutures, as to render it impossible to separate them without the knife. Whether this strong adhesion was natural, or the effect of disease, I am unwilling to decide positively, having never before seen the skull of an infant raised. Besides the disproportionate heat about the head, there were so many other symptoms indicating an affection of the brain, as to induce the family and the two attending physicians to believe, that the seat of the disease was in the head. The parents the more readily adopted this opinion, as they had lost several children, affected in a similar manner, whose diseases had been considered by their physicians affections of the brain. The lungs were perfectly collapsed and natural. Not the slightest adhesion could be found. The pericardium was discoloured, and contained about an ounce and a half of sero-purulent fluid. The whole surface of the heart was covered with a flaky purulent matter. The substance of the heart appeared natural. In the ventricles were formed what

I shall

I shall call polypi, for want of a more scientific name. That these had been formed previous to the child's death, I have but little doubt, as they had considerable strength of texture, and adhered so firmly to the *columnæ carneæ*, that they could not be separated without tearing their substance. The contents of the abdomen were all natural except the liver, which was very large in proportion to the other viscera. The gentlemen present were of opinion, that this was a morbid enlargement of the liver. It struck me as the "sanguineous engorgement" of the liver, mentioned by M. Corvisart, as frequently occurring in diseases of the heart from an obvious cause: an incision into its substance corroborated the opinion.—Do enlargements of the liver in affections of the brain proceed from the same cause? Is the circulation in some affections of the brain rendered slow and intermitting, by a direct sympathy between the heart and an oppressed brain, or only through the instrumentality of the lungs? What is the reciprocal influence of the brain and heart upon each other in disease and in health? We see patients with diseases of the latter, subapoplectic for days and even weeks before death, as happened in a case I witnessed the last spring. Does the enlargement of the liver, in our case, explain the reason why the child preferred the posture before-mentioned? A transudation of bile had given a part of the arch of the colon a deep bilious tinge. Absent on a journey, I did not see the patient until the day but one before its death. From the symptoms and history of the case, I was of opinion that the principal seat of the disease was within the thorax. Its precise character I could not determine. I advised the application of a blister upon the breast, and the use of the tincture of foxglove, which seemed to give a temporary relief.* The disease of the head, if any such existed, I considered secondary, knowing that the brain, the heart, and the lungs may be all secondarily affected, when the principal seat of disease is in either, and also that they may be similarly affected when the primary seat of the disease is in the skin.

The nature of diseases of the heart, together with their causes and connections with diseases in other parts, have become a very interesting subject of medical investigation. It is not long since the faculty, in general, have begun to suspect them; and for the most part they are, at present, considered incurable. But when we recollect how many other diseases have been so considered, which are now very ma-

* In diseases of the heart, I have seen great relief, and even the disease apparently kept at bay, by the foxglove alone, or by a combination of it with squills, soap, opium, and calomel in form of pills.

nageable, nageable, we must not despair of finding a cure for this formidable and most distressing class of diseases. By ascertaining their remote causes, may not physicians be able to *prevent* them? By learning to detect and distinguish their proximate causes in their earliest stages, or by tracing their connection with other diseases, may they not discover the clue which leads to a successful method of treatment? The epidemic which prevailed among us last winter and spring, was very successfully treated by emetics, sudorifics, epispastics,† and a judicious application of external warmth; and, from observation, and some dissections I have had an opportunity to make, I am convinced, that the *heart was more or less the organ principally affected, in almost every severe case.* As the same torpor upon the surface, coldness of extremities, clammy sweat, small, frequent, and often irregular or intermitting pulse exist in many, if not all the diseases of the heart, may we not, reasoning from analogy, infer that similar remedies may be useful in a generality of those cases? I have a patient now under my care, labouring under a chronic disease of the heart, who has been invariably relieved by the means above mentioned. Dilatations of the heart or aorta, ossifications of the valves, or any other cause of obstruction to the blood in its passage through the pulmonary or aortic systems, produce strong palpitations of the heart, and irregular or intermitting pulse, while in simple inflammation of the heart, its irritability being increased, a smaller quantity of its natural stimulus causes it to act; hence the very small and frequent pulse which accompanies that state of disease.—*New-England Journal.*

*Charles-Town, N. H. Sept.* 1, 1813.

---

*For the Medical and Physical Journal.*

### *Opinions concerning the Case of Joanna Southcott.*

YOU will readily conceive our anxiety for news on our first return to old England; but, whilst we were waiting with eagerness for something about the Princess of Wales, or the Princess Charlotte, nothing resounded on our deck but that a boy had died with child undelivered, and that a virgin

---

* Having observed that no patient died where a strangury had been produced by cantharides, I was disposed to give it internally, when a free external use did not bring on a strangury. The practice succeeded perfectly. By producing a strangury, we made a diversion in favour of parts more immediately essential to life. The strangury was distressing to bear, but nothing was to be feared from it, it being under the controul of medicine.

